# Secreting retroperitoneal latero-aortic paraganglioma revealed by acute abdominal pain: a case report

**DOI:** 10.11604/pamj.2023.44.39.24768

**Published:** 2023-01-19

**Authors:** Idriss Ziani, Ahmed Ibrahimi, Omar Bellouki, Fouad Zouidia, Hachem El Sayegh, Lounis Benslimane, Yassine Nouini

**Affiliations:** 1 Department of Urological Surgery A, Ibn Sina University Hospital, Faculty of Medicine of Rabat, Mohammed V University in Rabat, Rabat, Morocco,; 2 Department of Anatomopathology and Cytology, Ibn Sina University Hospital, Faculty of Medicine of Rabat, Mohammed V University in Rabat, Rabat, Morocco

**Keywords:** Paraganglioma, abdominal pain, retroperitoneal, lateral aortic, case report

## Abstract

We report the case of a retroperitoneal paraganglioma in a 35-year-old man discovered by abdominal pain. The abdomino-pelvic computed tomography (CT) showed a retro-peritoneal latero-aortic mass compatible with a paraganglioma, confirmed by the 24-hour urinary metanephrines and normetanephrines test. After an adequate pharmacological preparation, we decide to perform a laparoscopic resection of the paraganglioma. The surgery went without any complication. Blood pressure and urinary catecholamines were normal after the surgery. Our observation presents the particularity of the mode of discovery which is atypical, as well as the particularity of the therapeutic management, which is the laparoscopic resection of the mass.

## Introduction

Paraganglioma, also called extra-adrenal pheochromocytoma, is a neuroendocrine tumor that arises from ectodermal cells of the autonomic nervous system, or from chromaffin tissues remains. The adrenal site is usual (90%), extra-adrenal localization is rare, representing only 10% of paragangliomas with an estimated incidence of 2 to 8 per million [1]. Secreting retroperitoneal paragangliomas represent 2% [2]. We report a new case of secreting retroperitoneal paraganglioma, discovered by a non-specific acute abdominal pain in a young patient. Laparoscopic resection was performed with an excellent result. The atypical clinical presentation and laparoscopic resection make the particularity of this case.

## Patient and observation

**Patient information**: a 35-year-old male with no medical history was seen at the emergency department for acute abdominal pain with moderate headache.

**Clinical findings**: on physical examination, we found a performance status of 0, examination revealed high blood pressure (180/90 mmHg) with tachycardia (heart rate: 120 beats per minute).

**Timeline of current episode**: the symptoms had been evolving for 8 months with a gradual exacerbation.

**Figure 1:**
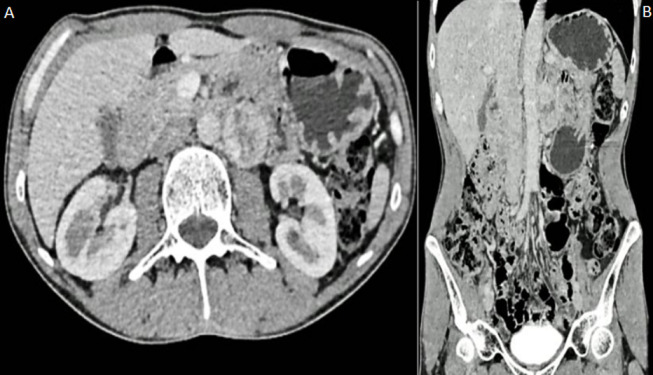
A) axial contrast-enhanced abdominal CT scan showing a left latero-aortic mass with heterogeneous enhancement; B) coronal contrast-enhanced abdominal CT scan showing the left latero-aortic mass

**Diagnostic assessment**: ultrasonography showed a 34 x 31 mm retro-peritoneal mass compatible with retro-peritoneal lymphadenopathy. Abdominal CT scan with contrast was performed and showed a 41 mm oval left latero-aortic mass with heterogeneous enhancement (central hypodensity compatible with necrosis with peripheral contrast enhancement). The mass was in intimate contact with the aorta. This description was compatible with paraganglioma or metastatic lymphadenopathy (Figure 1). A 24-hour urinary catecholamines test confirmed the diagnosis of secreting retroperitoneal paraganglioma with increased urinary normetanephrine levels (3220 nmol/24h) and normal metanephrine levels (64 nmol/24h). Blood pressure was controlled using a calcium channel blocker. A thoraco-abdominal-pelvic CT scan was performed and didn´t show any metastasis.

**Diagnosis**: considering the data from clinical, radiological and biological examinations, the diagnosis retained is a retroperitoneal latero-aortic paraganglioma secreting.

**Prognostic characteristics**: if the surgical resection is complete, the prognosis remains good with a rapid improvement in clinical signs, in particular arterial hypertension.

**Figure 2:**
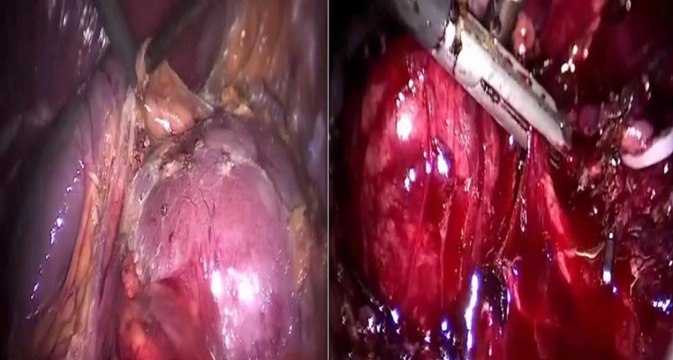
laparoscopic intraoperative image

**Therapeutic intervention and follow-up**: after anesthetic and cardiac assessment, our patient underwent a laparoscopic resection of the mass which was in intimate contact with the aorta inside, the renal pedicle above and the left ureter outside (Figure 2).

**Figure 3:**
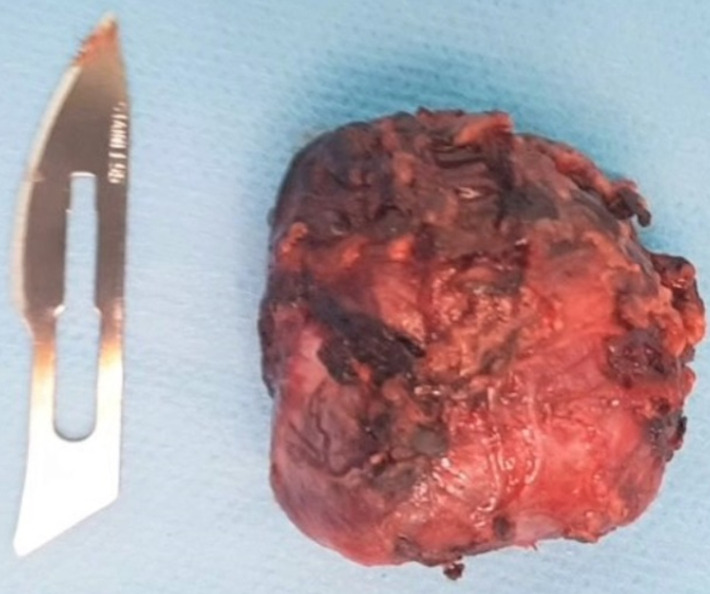
retroperitoneal paraganglioma after its resection and extraction

**Figure 4:**
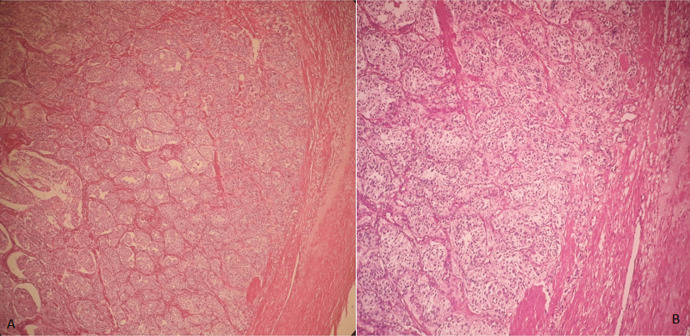
microscopic image; A) Gx40; B) Gx100 tumor growth well inscribed in the tumor organization in the form of small nests of large cells with clear cytoplasm

**Follow-up and outcome of interventions**: after surgery, the patient returned immediately to normal blood pressure and no longer needed to take antihypertensive therapy. Also, urinary normetanephrine levels were back to normal 1 month after surgery. At the anatomopathological level, the excision was complete; macroscopically it is a nodule of 3.3 X 0.5 cm of white-grayish aspect, well encapsulated with some bleeding reshuffle (Figure 3). Histologically, it is a tumor proliferation composed of areas, cords and clusters arranged around vascular structures. Realizing a neuroendocrine architecture, the tumor cells are large and have a finely granular eosinophilic cytoplasm with round nucleic nuclei, compatible with the histology of a paraganglioma (Figure 4).

**Patient perspective**: satisfied with the minimally invasive treatment without a large incision, clear improvement in clinical symptoms with normalization of biological assessment.

**Informed consent**: an informed, dated and signed consent was obtained from the patient allowing the said exploration to be carried out.

## Discussion

Primary retroperitoneal tumors in adults represent a highly heterogeneous group; more than 80% of these tumors are malignant [3]. Paraganglioma is a rare tumor that is developed from the ectodermal cells of the autonomic nervous system, or from remaining chromaffin tissues found along the axial skeleton and in the adrenal medulla [4]. Retroperitoneal paraganglioma is a rare entity [5]. Only two percent of paragangliomas are retroperitoneal [2]. The most common presentation of pheochromocytoma or paraganglioma is hypertension. However, pheochromocytoma is responsible for only 0.5% of secondary hypertension [5]. The classic symptoms including headaches, palpitations and profuse sweating are found in almost 90% of cases [6]. The other symptoms are less specific: chest and abdominal pain, anxiety, tremors, pallor and digestive disorders [5]. Almost 40% of paragangliomas are non-secretant, which explains the absence of the above-mentioned symptoms which makes the tumor hard to diagnose [7]. Abdominal ultrasound, computed tomography and MRI can help to diagnose the retroperitoneal mass. Paragangliomas can take many radiological aspects [8]. They can be made entirely of tissue, contain fat or calcify in places. Some tumors may necrosis with fluid hemorrhagic levels or give the appearance of a cystic mass with a fibrous capsule. Most often, tumors larger than 7cm are usually heterogeneous in density. There is no specificity in the type of enhancement [9]. MRI also has many aspects. In the absence of hypersecretion of catecholamines, a meta-iodine-benzyl-guanidine scintigraphy (MIBG) can also be performed as part of the diagnosis, however, this test would be positive in many non-functional paragangliomas [10,11]. On the other hand, it finds a preponderant place in post-operative surveillance where it allows the detection of recurrences or metastases [10,11].

The preoperative diagnosis is based on biological tests. The most sensitive and specific of these are the 24-hour urinary metanephrines and normetanephrines test with the free plasma metanephrines test. The plasma test offers a superior sensitivity with, however, less specificity than urinary tests. The chromogranin A test lacks specificity and sensitivity but may be useful to diagnose a non-functional paraganglioma and for follow-up [12]. The pre-anesthetic evaluation consists mainly of the assessment of cardiac function, since paraganglioma can be responsible of lethal adrenergic cardiomyopathy [13]. The management of paraganglioma requires multidisciplinary care. Surgical resection, when complete, remains the only curative treatment [14]. It allows survival rates of 75 and 45% at five and ten years, respectively [14]. To treat latero-aortic paraganglioma, surgical resection is usually performed. Laparoscopic resection of retroperitoneal paraganglioma is considered difficult, and the open transperitoneal approach remains the standard partic surgical procedure in most case [14]. In addition, surgery must be performed with care in an experienced center due to the serious hemodynamic side effects of the catecholamines released during handling. Therefore, there is currently no definitive opinion on the indications for laparoscopic surgery or established laparoscopic procedures. Currently, only a few studies have reported the use of this technique [7]. Surgical resection is still the gold standard therapy for retroperitoneal paraganglioma, due to their high risk of malignancy [10]. Radiotherapy is indicated as an analgesic to manage pain due to spinal metastasis or postoperatively to neutralize any tumor remains [4,10]. Chemotherapy can be considered in metastatic tumors and generally includes a combination of Dacarbazine, Vincristine and Cyclophosphamide [4,10]. In our observation, a complete resection of the tumor was performed laparoscopically without any complications. Biological disorders and blood pressure returned to normal after surgery.

## Conclusion

Retro-peritoneal paragangliomas are rare, the presentation of these tumors is not always specific. Their management must be multidisciplinary, surgical resection represents the only potentially curative treatment, it can be carried out by open or laparoscopic surgery in a specialized center; in case of incomplete resection the prognosis is reserved and the treatment is completed by radio or chemotherapy.
